# Gestational dating by metabolic profile at birth: a California cohort study

**DOI:** 10.1016/j.ajog.2015.11.029

**Published:** 2016-04

**Authors:** Laura L. Jelliffe-Pawlowski, Mary E. Norton, Rebecca J. Baer, Nicole Santos, George W. Rutherford

**Affiliations:** a Department of Epidemiology and Biostatistics, University of California, San Francisco School of Medicine, San Francisco, CA; b Department of Obstetrics, Gynecology, and Reproductive Sciences, University of California, San Francisco School of Medicine, San Francisco, CA; c Department of Pediatrics, University of California, San Diego School of Medicine, La Jolla, CA; d Global Health Sciences, University of California, San Francisco, San Francisco, CA

**Keywords:** acylcarnitines, amino acids, galactose-1-phosphate-uridyl-transferase, gestational dating, metabolic, metabolomics, preterm birth, thyroid-stimulating hormone, 17-hydroxyprogesterone

## Abstract

**Background:**

Accurate gestational dating is a critical component of obstetric and newborn care. In the absence of early ultrasound, many clinicians rely on less accurate measures, such as last menstrual period or symphysis-fundal height during pregnancy, or Dubowitz scoring or the Ballard (or New Ballard) method at birth. These measures often underestimate or overestimate gestational age and can lead to misclassification of babies as born preterm, which has both short- and long-term clinical care and public health implications.

**Objective:**

We sought to evaluate whether metabolic markers in newborns measured as part of routine screening for treatable inborn errors of metabolism can be used to develop a population-level metabolic gestational dating algorithm that is robust despite intrauterine growth restriction and can be used when fetal ultrasound dating is not available. We focused specifically on the ability of these markers to differentiate preterm births (PTBs) (<37 weeks) from term births and to assign a specific gestational age in the PTB group.

**Study Design:**

We evaluated a cohort of 729,503 singleton newborns with a California birth in 2005 through 2011 who had routine newborn metabolic screening and fetal ultrasound dating at 11–20 weeks’ gestation. Using training and testing subsets (divided in a ratio of 3:1) we evaluated the association among PTB, target newborn characteristics, acylcarnitines, amino acids, thyroid-stimulating hormone, 17-hydroxyprogesterone, and galactose-1-phosphate-uridyl-transferase. We used multivariate backward stepwise regression to test for associations and linear discriminate analyses to create a linear function for PTB and to assign a specific week of gestation. We used sensitivity, specificity, and positive predictive value to evaluate the performance of linear functions.

**Results:**

Along with birthweight and infant age at test, we included 35 of the 51 metabolic markers measured in the final multivariate model comparing PTBs and term births. Using a linear discriminate analyses-derived linear function, we were able to sort PTBs and term births accurately with sensitivities and specificities of ≥95% in both the training and testing subsets. Assignment of a specific week of gestation in those identified as PTBs resulted in the correct assignment of week ±2 weeks in 89.8% of all newborns in the training and 91.7% of those in the testing subset. When PTB rates were modeled using the metabolic dating algorithm compared to fetal ultrasound, PTB rates were 7.15% vs 6.11% in the training subset and 7.31% vs 6.25% in the testing subset.

**Conclusion:**

When considered in combination with birthweight and hours of age at test, metabolic profile evaluated within 8 days of birth appears to be a useful measure of PTB and, among those born preterm, of specific week of gestation ±2 weeks. Dating by metabolic profile may be useful in instances where there is no fetal ultrasound due to lack of availability or late entry into care.

## Introduction

Accurate gestational dating is a critical component of obstetric and newborn care. During pregnancy, gestational age informs the scheduling and management of clinical visits and laboratory testing, determination of the appropriateness of fetal growth, management and timing of delivery, evaluation of the pregnancy as being at risk for preterm and/or postterm delivery, and the application of interventions including, for example, the use of antenatal corticosteroids and magnesium for neuroprotection.^1^, ^2^, ^3^, ^4^ In the newborn period, gestational dating informs critical decisions around resuscitation (particularly in newborns born around the limits of viability) and is essential for tracking growth and neurodevelopmental function.^5^, ^6^

Accurate gestational dating is also important for establishing population-level rates of preterm birth (PTB). Measuring and tracking this rate across time is essential for establishing baselines and for resource planning aimed at reducing these outcomes and their associated burden within and across populations. While first-trimester ultrasound is recognized as the best method to establish gestational age for most pregnancies, ultrasound dating becomes less reliable as gestation progresses.^7^, ^8^, ^9^, ^10^, ^11^, ^12^, ^13^, ^14^, ^15^, ^16^, ^17^, ^18^ Prenatal ultrasound dating is also available only in sites with the resources to purchase the equipment and hire trained technicians. As such, prenatal ultrasound is not available in some rural and low- and middle-income country settings.^19^

In the absence of early ultrasound, many clinicians rely on last menstrual period (LMP) for gestational dating. LMP can be unreliable given that a number of factors can influence dating including poor recall, irregular cycle length, and bleeding in early pregnancy.^12^, ^16^, ^20^, ^21^ Symphysis-fundal height (SFH) >20 weeks is also used for pregnancy dating, but is often inaccurate due to factors such as multiple pregnancy, maternal size, polyhydramnios and oligohydramnios, and fetal growth restriction.^19^, ^22^

When no other measures of gestational dating are available (eg, fetal ultrasound, LMP, SFH), other measures used for gestational dating at birth include the Dubowitz method (which incorporates 34 physical and neurological assessments),^23^, ^24^ the Ballard method (which uses 6 physical and neurological measures taken within 30 and 42 hours of age),^25^ and the New Ballard score (which uses an expanded list of features over the original Ballard method).^26^ While all of these measures provide useful information when no other data are available, like with use of LMP and SFH, these methods have been shown to be less accurate when there is intrauterine growth restriction.^27^, ^28^, ^29^

The recognized need for a reliable alternative gestational dating method in instances where there is no first- or second-trimester ultrasound has led to an increased focus on identifying new dating methods and proxies that can be used during pregnancy or at the time of birth. Recent years have seen a punctuated effort in this area with the initiation of a number of high-risk, high-reward projects funded through the Bill and Melinda Gates Foundation^30^ and a recent call by the National Institutes of Health for more accurate tools to assess gestational age.^31^

In response to this call for newer dating methods, in this study we hypothesized that metabolic markers measured as part of routine newborn screening for treatable inborn errors of metabolism could be used to build a population-level metabolic dating algorithm that is robust despite intrauterine growth restriction. Specifically, we hypothesized that when considered in combination with newborn characteristics, metabolic markers would be able to differentiate PTBs (<37 weeks) from term births and to assign a specific gestational age within a margin of 2 weeks. If a metabolic dating tool were developed, it could be used broadly in high-income countries with existing newborn screening programs where no fetal ultrasound scan was done and could also be used by researchers and policy makers in other settings where newborn specimens are available for testing either retrospectively or prospectively to establish population-level baseline rates of PTB. Such a tool could gain further acceptance in countries without newborn screening through the use of miniature or hand-held mass spectrometers^32^ or via the translation of findings into a clinical assay that does not require the use of a mass spectrometer.

While our hypothesis that markers used for newborn screening could also be used for gestational dating purposes is novel, the work is well supported by previous studies demonstrating that many of these routinely collected markers (eg, acylcarnitines, amino acids, thyroid-stimulating hormone [TSH]) are related to PTB and to week of gestation and have heritable components that are robust in small-for-gestational-age (SGA) infants.^33^, ^34^, ^35^, ^36^, ^37^, ^38^, ^39^, ^40^, ^41^, ^42^, ^43^ In the present study, we developed and evaluated a metabolic dating algorithm using a large and diverse sample of California newborns who had ultrasound dating between 11–20 weeks of gestation. We focused specifically on the capacity of markers to differentiate PTBs from term births and to assign a specific gestational age in the preterm group.

## Materials and Methods

We evaluated whether biomarkers collected as part of routine newborn screening for treatable inborn errors of metabolism could be used to create a metabolic dating algorithm in a cohort of 729,503 singleton newborns born in California in 2005 through 2011. All of these newborns had routine metabolic screening performed through the California Newborn Screening program using a heel-stick blood draw between 12 hours and 8 days after birth. All babies had a mother who had ultrasound dating from 11–20 weeks of gestation, had a linked birth certificate and hospital discharge record, and were born at 22–44 completed weeks of gestation (sample selection included in Figure). For study purposes, we randomly divided the final cohort into a training subset with 547,127 babies (75% of total) and a testing subset with 182,376 babies (25% of total). This division allowed for an unbiased estimate of model performance given that the testing set was not part of the sample in which the initial model was built.

Date of birth, birthweight, gender, and race/ethnicity were derived from the birth cohort files, which are linked birth certificate and hospital discharge files obtained from the California Office of Statewide Health Planning and Development. Age at testing in hours after birth, blood-spot analyte measurements, and information about whether the infant had been on total parenteral nutrition (TPN) between birth and the time of testing was obtained from newborn screening records. We were able to identify newborns with fetal ultrasound dating from 11–20 weeks of gestation by examining linked newborn and prenatal screening records. We computed days of gestation at birth by comparing the estimated date of delivery based on ultrasound findings in the prenatal screening records to the birth date on the linked birth certificate and hospital discharge records. In California, the prenatal and newborn screening programs are nested within the same division of the California Department of Public Health (the Genetic Disease Screening Program). This nesting allows for routine linkage of prenatal and newborn screening records. The prenatal and newborn screening data used in this study were obtained from the California Biobank Program. Details regarding the California Newborn Screening Program and the California Prenatal Screening Program have been described in detail elsewhere.^44^, ^45^

All markers evaluated in the study were tested by the California Department of Public Health’s Genetic Disease Laboratory as part of routine screening for treatable inborn errors of metabolism using dried blood specimens collected by heel-stick at birth hospitals from 12 hours to 8 days after birth. Free carnitine, acylcarnitines (C-2, C-3, C-3DC, C-4, C-5, C-5:1, C-5DC, C-6, C-8, C-8:1, C-10, C-10:1, C-12, C-12:1, C-14, C-14:1, C-16, C-16:1, C-18, C-18:1, C-18:2, C-18:1OH), and amino acids (alanine, arginine, citrulline, glycine, methionine, ornithine, phenylalanine, proline, 5-oxoproline, tyrosine, valine) were measured by standardized tandem mass spectrometry. TSH and 17-hydroxyprogesterone (17-OHP) were measured using high-performance liquid chromatography. Galactose-1-phosphate-uridyl-transferase was measured using a fluorometric enzyme assay. The study also utilized a number of ratios commonly used in screening (C-14:1/C-12:1, C-1/C-2, C-8/C-10, free carnitine/(C-16 + C-18:1), arginine/ornithine, citrulline/arginine, leucine/alanine, leucine/isoleucine, ornithine/citrulline, and phenylalanine/tyrosine). We transformed biomarker values and ratios using natural logarithms to normalize distribution across all markers.

Analyses first focused on evaluating the association among maternal characteristics, markers, and PTB in the training subset using simple bivariate logistic regression and related odds ratios and 95% confidence intervals (CI). We used multivariate backward stepwise regression for final model building with entry at *P* < .40 based on bivariate analyses and removal at *P* < .05. TPN was included in multivariate models regardless of observed *P* values given the demonstrated relationship between TPN and some markers tested as part of routine screening (eg, some acylcarnitines and amino acids)^40^, ^41^, ^46^, ^47^ and our desire to identify markers with a robust association with gestational age despite TPN status. Characteristics and markers remaining in the final TPN-adjusted multivariate model were used to create a linear discriminate analysis (LDA)-derived linear function that we used to sort PTBs and term births. Markers were also leveraged to create an LDA-derived linear function for specific week of gestation in those identified as preterm. We evaluated the performance of the linear functions in both the training and testing subsets using sensitivity, specificity, and positive predictive values (PPVs) and their 95% CIs. We evaluated performance in all births and in subsets grouped as SGA (<10th percentile weight for gestational age), appropriate for gestational age (10th–90th percentile weight for gestational age), and large for gestational age (>90th percentile weight for gestational age) based on published US norms.^48^ Week-specific LDA-derived linear functions were evaluated by their capacity to assign week of gestation in those identified as preterm compared to ultrasound-dated week. We further examined the modeled rates of PTB based on the metabolic dating algorithm compared to ultrasound-dated week.

We performed all analyses using software (SAS, Version 9.3; SAS Institute Inc, Cary, NC). Methods and protocols for the study were approved by the Committee for the Protection of Human Subjects within the Health and Human Services Agency of the State of California (protocol no. 12-09-0702).

## Results

The study cohort included 729,503 singleton infants (20.2% of the total population of singletons in these birth years [n = 3,617,727]). This number represented those with ultrasound results and linked birth and hospital discharge records who had gestational age from 22–44 weeks (Figure). Race/ethnicity and gender was similar to that of all births during this time period^49^ wherein most newborns were Hispanic (47.1%) or non-Hispanic white (32.0%) with more male than female births (51.1% vs 48.6%). Nine in 10 newborns had newborn screening conducted within 3 days (Table 1).

Along with a number of infant characteristics, 49 of the 51 metabolites and metabolite ratios measured were associated with PTB in crude logistic regression analyses (all except C-5DC and C-18) (Supplementary Table 1). Male gender, race/ethnicity, and 14 metabolic markers were removed from final multivariate logistic models using stepwise methods given *P* values >.05. Hour of age at collection, birthweight, and 35 metabolic markers were included in the final multivariate logistic regression model (Table 2). When a LDA-derived linear function was built based on these training set multivariate logistic results, we found that these characteristics and markers were able to identify PTBs with >95% accuracy in the training and testing subsets (sensitivity 99.5% [95% CI, 99.5–99.6%], specificity 98.8% [95% CI, 98.8–98.9%] in the training subset; sensitivity 99.5% [95% CI, 99.4–99.6%], specificity 98.9% [95% CI, 98.8–98.9%] in the testing subset). Findings were robust across SGA, appropriate-for-gestational-age, and large-for-gestational-age babies (sensitivity and specificity across all groups ≥94.9%). PPVs tended to be >85% across most groupings with the exception of the SGA group where PPVs were >66% (66.0% [95% CI, 64.6–67.2%] in the training set, 66.7% [95% CI, 64.4–68.9%] in the testing subset) (Table 3). Assignment of a specific week of gestation in those identified as preterm resulted in the correct assignment ±2 weeks in 89.8% of all newborns in the training set and 91.7% of newborns in the testing subset (Table 4).

Our final metabolic dating algorithm relied first on sorting PTBs and term births using the LDA-derived linear function in Supplementary Table 2, and then, assigning weeks of gestation to those identified as preterm using the linear function in Supplementary Tables 3 and 4. When this algorithm was tested against prenatal ultrasound at 11–20 weeks, we found that it calculated the incidence of PTB <37 weeks as 7.15% vs 6.11% in the training set and 7.31% vs 6.25% in the testing subset, the rate of PTB <32 weeks as 0.71% vs 0.53% in the training set and 0.71% vs 0.51% in the testing subset, and PTB 32–36 weeks as 6.44% vs 5.58% in the training set and 6.60% vs 5.74% in the testing subset (Table 5).

## Comment

Using birthweight, age at testing, and a number of the markers measured as part of routine newborn screening for treatable inborn errors of metabolism within 8 days of life (acylcarnitines, amino acids, TSH, 17-OHP, and galactose-1-phosphate-uridyl-transferase), we were able to build a metabolic dating algorithm that was able to consistently sort PTBs from term births with approximately ≥95% sensitivity and specificity. Among newborns identified as preterm, we were able to assign a gestational week that was within 2 weeks of gestational age determined by ultrasound in about 90% of cases. PTB rates using metabolic dating were within a range of about 1% of those generated using ultrasound.

While no other published study that we are aware of has used the combination of these markers for gestational dating, our findings are in agreement with other studies that have demonstrated an association between PTB and many of the individual biomarkers studied including those that have found that differences remain after accounting for SGA and feeding status.^33^, ^34^, ^35^, ^36^, ^37^, ^38^, ^39^, ^40^, ^41^, ^42^, ^43^ Like other investigators we observed significant differences between PTBs and term births in free carnitine^33^, ^39^; short-, medium-, and long-chain acylcarnitines^33^, ^39^, ^40^, ^41^, ^43^; amnio acids^39^, ^40^, ^41^, ^43^; TSH^35^, ^37^, ^50^; and 17-OHP.^47^, ^51^, ^52^ Although it is unclear what specifically underlies the differences observed and why they appear useful for gestational dating, it appears that both etiological and maturational underpinnings may exist that are marker specific. For instance, 2 of the acylcarnitines (C-8:1 and C-18:2) included in the final predictive model have been shown to be associated with maternal preeclampsia in studies that have examined maternal serum during pregnancy and newborn bloodspots.^47^, ^53^ Reasons for these associations have been hypothesized as being related to abnormalities in fatty acid oxidation in the mother, the baby, or both suggesting an etiological link. While other work has found that C-8:1 and C-18:2 are not as closely tied to specific gestational age among those born preterm, other acylcarnitines in our final model including C-4, C-5, and C-6 are.^34^ This latter pattern suggests that these marker patterns may be more closely tied to maturation. Similar suspected links to fatty acid metabolism and maturation have been discussed at length with respect to other acylcarnitines and amino acids included in our final model as well as TSH and 17-OHP.^36^, ^38^, ^40^, ^41^, ^42^, ^44^, ^51^, ^52^

For the most part, studies that have evaluated methods of dating absent ultrasound have focused on LMP and SFH during pregnancy^12^, ^16^, ^19^, ^20^, ^21^, ^22^ and the Dubowitz, Ballard, and the New Ballard methods at birth.^23^, ^24^, ^25^, ^26^ In general, all of these measures have some difficulties in approximating gestational age, often for seemingly different reasons. With LMP it appears that there is a tendency to date a pregnancy later, which then leads to a greater tendency to label a birth as being preterm^54^; with SFH, the Dubowitz, and the older and New Ballard methods, underestimates often result and appear to be more closely tied to problems with dating when there is intrauterine growth restriction.^19^, ^22^, ^27^, ^28^, ^29^ For example, in a recent study of all US birth certificates from 2012, Duryea and colleagues^54^ found that use of LMP instead of ultrasound-derived best obstetric estimate led to an overall overestimate in the national PTB rate of >1.9% and overestimates of PTB rates in teenagers and non-Hispanic blacks of >3%. With respect to use of the Dubowitz, Ballard, and the new Ballard, studies have found that these measures tend to perform particularly poorly in babies born preterm with agreement with ultrasound ±2 weeks of as little as 55%.^27^, ^28^, ^29^

The metabolic dating algorithm presented here appears to represent an improvement over dating by LMP, SFH, Dubowitz, Ballard, or the New Ballard given that it was able to identify babies born preterm with >99% sensitivity and specificity and had associated PTB rates that were within 1% of those determined using ultrasound measures. Further support for this assessment is demonstrated by its capacity to assign gestational age within 2 weeks in 90% of those identified as preterm. Such findings require careful replication, and it should be noted that even with replication, it is unclear how metabolic dating might be used. In the United States and developed countries, there would likely be opportunity to leverage these data in instances where ultrasound dating was not done given routine testing of these markers. Where the testing of the markers is already being done as part of routine newborn screening for treatable inborn errors of metabolism, translation could be accomplished, for example, at hospitals or clinics through an online application. It is also possible that this algorithm could be run routinely for all newborns through partnership with newborn screening programs. In lower resource settings where routine measurement is not done and where mass spectrometry technology is often not available, use of this algorithm might be more aptly used for retrospective baselining of PTB rates using banked serum specimens. Use of miniature or hand-held mass spectrometers^32^ or translation of findings into a clinical assay that does not require mass spectrometry could potentially lead to wider prospective use in those settings if the algorithms were found to replicate.

Important strengths of the present study include the use of a large and diverse sample of newborns who had fetal ultrasound dating from 11-20 weeks. Analyses also benefited from the availability of birthweight and gestational age, which allowed for the evaluation of performance in the face of intrauterine growth restriction. Both of these strengths should be considered in tandem with limitations. For example, while our sample was representative of the diverse race/ethnic makeup of all California births, it is possible that other factors like poverty, nutrition, and access to care could have affected access to an early ultrasound. Further follow-up in target populations is essential to assess performance. With respect to the strength of being able to look at patterns among the SGA group, it is important to note that while sensitivities and specificities were >95% across training and testing subsets, PPVs were around 66.0%. This finding means that about 33% of SGA term babies were wrongly coded as preterm when in fact they were term. This finding lends further support to the need for replication and suggests a need to work toward even better performance.

## Conclusion

In combination with birthweight and hours of age at test, metabolic profile evaluated within 8 days of birth appears to be a useful measure of PTB and, among those born preterm, of specific week of gestation ±2 weeks. Dating by metabolic profile may be useful in instances where there is no dating by prenatal ultrasound due to lack of availability or late entry into obstetric care.

## Figures and Tables

**Supplementary Table 1:** Logistic regression: crude results, risk of preterm birth by specific bivariate target categorical variables and by per-natural log unit increase in target continuous variables

	OR	95% CI	*P* value
Total parenteral nutrition^a^	1.000	1.000–1.000	.326
Male gender^a^	1.215	1.188–1.243	<.001
Race/ethnicity^a^			
White	0.982	0.960–1.005	.129
Hispanic	1.241	1.213–1.268	<.001
Black	1.134	1.072–1.201	<.001
Asian	0.925	0.894–0.958	<.001
Other	1.046	1.019–1.074	<.001
Hour at collection^b^	1.032	1.031–1.032	<.001
Birthweight^b^	0.997	0.997–0.997	<.001
Metabolites^b^			
FC	2.193	2.137–2.251	<.001
Acylcarnitines			
C-2	1.183	1.143–1.225	<.001
C-3	3.024	2.939–3.111	<.001
C-3DC	0.650	0.631–0.669	<.001
C-4	2.369	2.312–2.428	<.001
C-5	7.709	7.509–7.914	<.001
C-5:1	1.162	1.146–1.178	<.001
C-5DC	1.011	0.987–1.035	.3858
C-5OH	1.223	1.193–1.254	<.001
C-6	0.966	0.951–0.981	<.001
C-8	1.140	1.120–1.161	<.001
C-8:1	1.424	1.393–1.456	<.001
C-10	0.686	0.672–0.702	<.001
C-10:1	1.343	1.317–1.370	<.001
C-12	0.632	0.620–0.645	<.001
C-12:1	0.556	0.546–0.566	<.001
C-14	0.862	0.837–0.889	<.001
C-14:1	0.797	0.780–0.815	<.001
C-14OH	0.851	0.839–0.863	<.001
C-16	0.296	0.287–0.306	<.001
C-16:1	0.506	0.495–0.518	<.001
C-16OH	0.831	0.819–0.843	<.001
C-18	0.974	0.942–1.006	.106
C-18OH	0.905	0.890–0.915	<.001
C-18:1	1.112	1.073–1.151	<.001
C-18:1OH	0.902	0.890–0.915	<.001
C-18:2	4.743	4.635–4.853	<.001
C-14:1/C-12:1	1.799	1.761–1.838	<.001
C-1/C-2	3.323	3.219–3.429	<.001
C-8/C-10	1.202	1.185–1.219	<.001
FC/(C-16 + C-18:1)	3.144	3.067–3.223	<.001
Amino acids			
Alanine	0.424	0.408–0.440	<.001
Arginine	1.748	1.718–1.778	<.001
Citrulline	0.385	0.370–0.401	<.001
Glycine	0.436	0.417–0.456	<.001
Methionine	3.827	3.687–3.973	<.001
Ornithine	1.427	1.381–1.475	<.001
Phenylalanine	4.720	4.491–4.961	<.001
Proline	1.366	1.317–1.417	<.001
5-Oxoproline	0.949	0.933–0.965	<.001
Tyrosine	3.959	3.844–4.077	<.001
Valine	2.547	2.455–2.643	<.001
Arginine/ornithine	1.609	1.580–1.638	<.001
Citrulline/arginine	0.467	0.459–0.476	<.001
Phenylalanine/tyrosine	0.444	0.431–0.457	<.001
Leucine/arginine	11.787	11.359–12.232	<.001
Ornithine/citrulline	2.441	2.366–2.518	<.001
Leucine/isoleucine	8.077	7.770–8.395	<.001
Other markers			
TSH	0.496	0.489–0.504	<.001
17-OHP	3.888	3.814–3.964	<.001
GALT	0.479	0.457–0.501	<.001

*CI*, confidence interval; *FC*, free carnitine; *GALT*, galactose-1-phosphate-uridyl-transferase; *OR*, odds ratio; *TSH*, thyroid-stimulating hormone; *17-OHP*, 17-hydroxyprogesterone.

*Jelliffe-Pawlowski et al. Dating by metabolic profile: California. Am J Obstet Gynecol 2016*.

a Yes vs no

b Considered as continuous variable.

**Supplementary Table 2:** Linear discriminate for <37 weeks including markers and characteristics in final multivariate model (Table 2)^a^

Constant	–1638
Alanine	–18.24294
C-3	–10.58859
C-3DC	–37.34471
C-4	–3.91865
C-5	–7.79132
C-5:1	–4.97254
C-5DC	17.50509
C-6	–4.04150
C-8:1	–1.83483
C-10	–5.82510
C-12	–18.94594
C-12:1	–3.1822
C-14	–23.82009
C-14:1	–0.74261
C-16:1	–38.39710
C-18	–12.85971
C-18:1	–35.36104
C-18:2	–15.45137
FC	110.08731
Glycine	77.65724
Methionine	–8.43507
17-Hydroxyprogesterone	25.70541
Ornithine	–30.59164
5-Oxoproline	–5.62405
Proline	19.81972
C-14/C-12	0.98818
Citrulline/arginine	7.26629
FC/(C-16 + C-18:1)	–77.59400
Phenylalanine/tyrosine	48.51520
Ornithine/citrulline	10.20481
Galactose-1-phosphate-uridyl-transferase	125.71008
Thyroid-stimulating hormone	4.45184
Tyrosine	100.77265
Valine	–15.02337
Leucine/isoleucine	56.81949
Birthweight	–0.01154
Hour at collection	0.87703

*FC*, free carnitine.

*Jelliffe-Pawlowski et al. Dating by metabolic profile: California. Am J Obstet Gynecol 2016*.

a All markers and characteristics natural log transformed.

**Supplementary Table 3:** Linear discriminate for week of gestation among those <37 weeks based on <37 discriminate (weeks 22–29)^a^

	22	23	24	25	26	27	28	29
Constant	–932.50675	–953.2113	–944.19404	–948.56204	–955.93217	–959.50151	–976.27912	–982.90321
Alanine	8.28894	5.19942	5.113	4.62529	3.86256	4.22772	3.7845	3.93171
C-3	–16.57929	–18.46201	–17.87288	–17.76792	–17.71771	–17.82173	–17.44024	–17.698
C-3DC	–26.19027	–24.67952	–23.93584	–24.53703	–26.23626	–25.96805	–27.12178	–26.73299
C-4	–3.49777	–3.32801	–3.45246	–3.4786	–3.83773	–4.18724	–4.30985	–4.54618
C-5	–15.17012	–13.27513	–13.4379	–13.80958	–14.44197	–14.52518	–14.7515	–14.9885
C-5:1	–5.20145	–5.50342	–5.37255	–5.30278	–5.28731	–5.23548	–5.30954	–5.31411
C-5DC	8.61735	9.44106	9.35617	8.93827	9.2908	9.03588	9.6062	9.33133
C-6	–2.43769	–2.70927	–2.31853	–2.54648	–2.43333	–2.56419	–2.71891	–2.56632
C-8:1	–3.54416	–1.30691	–1.27859	–1.14956	–0.99902	–0.95415	–1.00536	–1.03539
C-10	–1.45075	–2.32381	–2.89901	–2.61843	–2.98864	–3.22778	–2.67265	–3.04453
C-12	–9.83389	–11.16881	–10.79162	–10.86977	–10.82765	–10.98124	–10.68184	–10.84405
C-12:1	–4.30408	–5.17368	–3.18408	–3.42082	–3.72526	–3.50739	–4.70966	–3.58627
C-14	–24.11108	–25.88159	–25.01019	–25.02206	–24.87757	–24.51515	–25.50242	–24.42464
C-14:1	6.93662	7.6508	5.2393	5.49597	6.31254	6.28035	7.70797	6.18614
C-16:1	–28.26956	–30.04201	–29.88644	–29.83031	–30.38756	–30.03092	–30.85866	–30.87226
C-18	–19.04334	–18.99306	–19.29755	–19.47758	–19.59952	–19.75949	–20.23575	–20.90977
C-18:1	–27.08947	–27.34848	–26.73875	–28.0138	–28.65903	–29.87428	–31.29109	–31.51176
C-18:2	–18.23344	–19.76523	–18.9327	–18.91284	–18.17538	–17.52888	–17.2471	–17.61357
FC	103.89573	110.25176	108.33635	110.84605	112.29876	113.22328	115.79517	117.10496
Glycine	64.78615	64.88581	64.06058	63.84417	64.26949	64.10494	64.27953	63.82198
Methionine	–27.9073	–24.10761	–23.41544	–24.82403	–24.14709	–24.0655	–23.77045	–23.05988
17-OHP	15.15918	15.89029	15.72505	15.39154	15.08971	14.81963	14.37009	14.15223
Ornithine	–21.02409	–20.63325	–19.16193	–18.51997	–18.09949	–18.38862	–18.35503	–18.47604
5-Oxoproline	–6.63031	–5.98036	–5.86063	–5.8677	–6.00961	–6.21009	–5.92026	–5.94451
Proline	13.25993	15.82941	15.36645	15.05716	15.88264	15.73205	15.9093	15.95879
C-14/C-12	–2.53122	–2.94236	–1.96468	–2.14515	–3.04039	–2.54535	–3.80081	–2.57918
Citrulline/arginine	11.59236	11.65395	11.79839	11.48146	11.66921	11.74594	11.6364	11.85715
FC/(C-16 + C-18:1)	–64.34898	–70.50515	–68.60503	–70.80802	–73.21485	–74.42308	–77.05317	–78.49802
Phenylalanine/tyrosine	29.38101	30.9779	30.30119	31.74303	32.59037	32.31711	33.66762	33.54525
Ornithine/citrulline	7.4646	5.17964	4.93853	4.94507	5.90052	6.37034	6.96901	7.54495
GALT	105.89469	106.88822	107.56674	107.74282	108.86221	109.89883	111.26397	112.24477
TSH	1.91686	1.15133	1.02702	1.31333	2.05631	2.44641	3.115	3.45602
Tyrosine	55.10445	57.34291	56.23898	57.21708	57.63742	56.9794	58.07309	57.79313
Valine	3.69641	2.35564	2.57893	2.91362	1.85609	1.83322	1.37229	1.6306
Leucine/isoleucine	42.61538	40.77523	40.2314	40.59463	39.63484	40.17964	39.87732	39.60262
Birthweight	0.0079	0.00755	0.0081	0.00791	0.0088	0.00932	0.00976	0.01033
Hour at collection	0.26349	0.29616	0.2839	0.28964	0.30761	0.31524	0.33132	0.33422

*FC*, free carnitine; *GALT*, galactose-1-phosphate-uridyl-transferase; *TSH*, thyroid-stimulating hormone; *17-OHP*, 17-hydroxyprogesterone.

*Jelliffe-Pawlowski et al. Dating by metabolic profile: California. Am J Obstet Gynecol 2016*.

a All markers and characteristicsnatural log transformed.

**Supplementary Table 4:** Linear discriminate for week of gestation among those <37 weeks based on <37 discriminate (weeks 30–36)^a^

	30	31	32	33	34	35	36
Constant	–988.50527	–995.75501	–1002	–1002	–998.81947	–996.26659	–999.34745
Alanine	4.2087	4.63183	5.28633	5.69558	6.14229	6.20216	6.44306
C-3	–17.60178	–17.7607	–17.77486	–17.69336	–17.76725	–18.13269	–18.62529
C-3DC	–27.68125	–28.25113	–28.9111	–29.53141	–29.88425	–29.73349	–29.39607
C-4	–4.55088	–4.7859	–4.83465	–4.98735	–5.0364	–5.05325	–5.07372
C-5	–15.09459	–15.19294	–15.34302	–15.54043	–15.88192	–16.02557	–16.2285
C-5:1	–5.35522	–5.42657	–5.44484	–5.46036	–5.51482	–5.55429	–5.5616
C-5DC	9.56253	9.27383	9.35232	9.76537	9.93317	9.92958	9.67189
C-6	–2.69388	–2.59329	–2.58995	–2.47167	–2.5003	–2.52172	–2.49855
C-8:1	–0.73882	–0.68425	–0.83416	–0.53371	–0.32432	–0.23999	–0.31901
C-10	–3.03439	–2.51854	–2.42259	–2.2638	–2.10738	–2.06029	–1.98061
C-12	–10.74325	–10.55858	–10.52381	–10.31629	–10.06991	–9.68766	–9.58632
C-12:1	–2.82098	–2.41691	–2.5843	–2.1284	–2.3772	–2.377	–2.19121
C-14	–24.35735	–23.68109	–23.44333	–23.23374	–23.24398	–23.37978	–23.54099
C-14:1	5.64052	4.48174	4.57604	3.82457	4.35081	4.89146	4.90526
C-16:1	–30.9545	–30.99005	–31.26494	–31.30972	–31.66705	–31.83462	–31.95655
C-18	–21.113	–21.37849	–21.61745	–22.16167	–22.88771	–23.13368	–23.03947
C-18:1	–31.58447	–32.71608	–32.61122	–32.51187	–31.55139	–31.52162	–31.83863
C-18:2	–17.28389	–17.43331	–17.59197	–17.57429	–17.87897	–17.89621	–17.89402
FC	117.35909	118.973	119.32926	119.78986	120.27383	120.61386	121.04239
Glycine	64.02354	63.93202	63.79536	63.68921	63.68449	63.92172	63.82196
Methionine	–23.72761	–23.35167	–23.21117	–23.02348	–22.96024	–22.85355	–22.81308
17-OHP	13.56281	13.24651	13.18203	13.05065	12.83415	12.50796	12.24516
Ornithine	–18.95869	–18.73249	–18.87447	–19.29801	–19.63549	–19.22495	–18.6176
5-Oxoproline	–6.05998	–6.02286	–6.10127	–5.89173	–5.96226	–5.97521	–5.95991
Proline	16.44024	16.18929	16.42513	16.9491	17.23069	17.42308	17.438
C-14/C-12	–2.14244	–1.57293	–1.70284	–1.41461	–1.60313	–1.86508	–1.86071
Citrulline/arginine	11.94376	12.07144	12.26846	12.53041	12.67593	12.7734	12.76036
FC/(C-16 + C-18:1)	–78.71474	–80.49481	–81.17256	–81.80405	–82.61718	–83.21782	–83.7118
Phenylalanine/tyrosine	34.56247	34.40066	33.90638	32.9183	32.63234	32.02879	31.70916
Ornithine/citrulline	8.34688	8.36896	8.79664	9.27236	9.64076	9.55739	9.27991
GALT	113.10771	113.38068	113.72742	113.67361	113.26317	113.05344	113.38014
TSH	3.58492	3.80202	3.95464	4.09603	4.33518	4.48642	4.56842
Tyrosine	58.82914	59.05708	58.79186	58.10647	57.76591	56.9272	56.30401
Valine	1.05805	0.91155	0.86614	0.63452	0.60383	0.52097	0.6643
Leucine/isoleucine	39.02383	38.99794	38.58841	38.14015	37.45876	37.2543	37.01122
Birthweight	0.0113	0.01203	0.01267	0.01367	0.01446	0.01535	0.01631
Age at collection	0.33723	0.33865	0.34199	0.34225	0.33639	0.32541	0.31429

*FC*, free carnitine; *GALT*, galactose-1-phosphate-uridyl-transferase; *TSH*, thyroid-stimulating hormone; *17-OHP*, 17-hydroxyprogesterone.

*Jelliffe-Pawlowski et al. Dating by metabolic profile: California. Am J Obstet Gynecol 2016.*

a All markers and characteristics natural log transformed.

**Figure:**
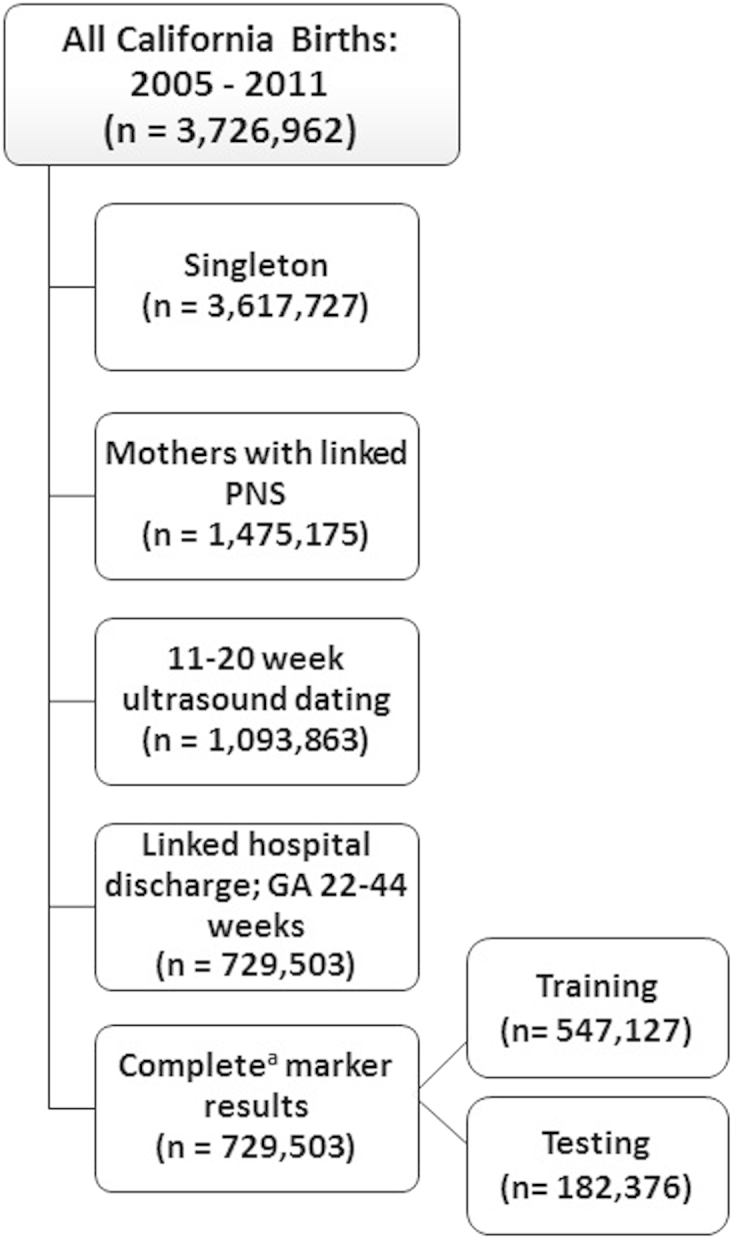
Sample selection ^a^Results present for 51 target markers and ratios measured between 12 hours and 8 days after birth as part of routine newborn screening. *GA*, gestational age; *PNS*, prenatal screening. *Jelliffe-Pawlowski et al. Dating by metabolic profile: California. Am J Obstet Gynecol 2016.*

**Table 1:** Newborn characteristics

	n = a	%
Sample	729,503	100.0
Race/ethnicity		
White, not Hispanic	233,729	32.0
Hispanic	343,388	47.1
Asian	90,219	12.4
Black	25,814	3.5
Other	36,307	5.0
Gender		
Male	372,689	51.1
Female	354,189	48.6
Completed gestation, wk^b^		
<32	3809	0.5
32–36	41,014	5.6
≥37	684,680	93.9
Weight for gestational age^c^		
<10th (SGA)	51,469	7.1
10–90th (AGA)	617,147	84.6
>90th (LGA)	60,887	8.4
H/d at testing		
12–24 h	262,323	36.0
2–3 d	397,301	54.5
4–5 d	51,076	7.0
7–8 d	18,803	2.6
Total parenteral nutrition	10,264	1.4

*AGA*, appropriate-for-gestational age; *LGA*, large-for-gestational age; *SGA*, small-for-gestational age.

*Jelliffe-Pawlowski et al. Dating by metabolic profile: California. Am J Obstet Gynecol 2016*.

a Value missing where sum for category does not equal total sample size

b Based on 11–20 wk fetal ultrasound

c Using Alexander et al.^48^

**Table 2:** Markers and characteristics in final <37 weeks’ model^a^

	^Adj^OR	95% CI
Acylcarnitines		
C-3	3.526	3.344–3.719
C-3DC	0.750	0.706–0.798
C-4	1.054	1.017–1.093
C-5	1.918	1.845–1.995
C-5:1	1.032	1.014–1.051
C-5DC	1.972	1.868–2.082
C-6	0.930	0.911–0.950
C-8:1	1.083	1.053–1.115
C-10	0.860	0.829–0.893
C-12	0.835	0.802–0.870
C-12:1	0.589	0.524–0.662
C-14	1.394	1.312–1.481
C-14:1	1.427	1.245–1.635
C16:1	1.160	1.097–1.226
C-18	1.383	1.279–1.496
C-18:1	1.692	1.527–1.876
C-18:2	1.192	1.143–1.243
Free carnitine	0.146	0.127–0.168
C-14:1/C-12:1	0.805	0.722–0.897
Free carnitine/(C-16 + C-18:1)	0.146	0.127–0.168
Amino acids		
Alanine	0.135	0.126–0.144
Glycine	1.399	1.299–1.508
Methionine	1.291	1.205–1.384
Ornithine	0.263	0.243–0.284
Proline	0.889	0.832–0.949
Tyrosine	7.940	7.128–8.845
Valine	0.566	0.525–0.611
5-Oxoproline	1.144	1.108–1.181
Leucine/isoleucine	3.020	2.729–3.342
Citrulline/arginine	0.849	0.827–0.873
Phenylalanine/tyrosine	2.235	2.022–2.470
Ornithine/citrulline	2.137	2.002–2.470
Other		
Thyroid-stimulating hormone	0.810	0.788–0.832
17-OHP	2.963	2.879–3.048
GALT	0.552	0.513–0.593
Birthweight^b^	0.997	0.997–0.997
Hour at test^b^	1.025	1.024–1.027

^*Adj*^*OR*, adjusted odds ratio; *CI*, confidence interval; *FC*, free carnitine; *GALT*, galactose-1-phosphate-uridyl-transferase; *17-OHP*, 17-hydroxyprogesterone.

*Jelliffe-Pawlowski et al. Dating by metabolic profile: California. Am J Obstet Gynecol 2016*.

a All variables natural log-transformed, associations *P* < .01 after adjustment for all other markers in model and for total parenteral nutrition

b Included as continuous variable.

**Table 3:** Performance of linear discriminate for <37 completed weeks’ gestation

	Training			Testing		
Sensitivity (95% CI)	Specificity (95% CI)	PPV (95% CI)	Sensitivity (95% CI)	Specificity (95% CI)	PPV (95% CI)
All	99.5%	98.9%	85.1%	99.5%	98.8%	85.2%
(99.5–99.6)	(98.8–98.9)	(84.7–85.4)	(99.4–99.6)	(98.8–98.9)	(84.6–85.8)
SGA^a^	99.9	95.1	66.0	100.0	95.0	66.7
(99.8–100.0)	(94.8–95.3)	(64.6–67.2)	(99.6–100.0)	(94.6–95.4)	(64.4–68.9)
AGA^a^	99.6	99.1	87.9	99.6	99.1	87.9
(99.5–99.7)	(99.1–99.1)	(87.5–88.2)	(99.5–99.7)	(99.0–99.1)	(87.2–88.5)
LGA^a^	95.4	99.7	89.3	94.9	99.7	89.4
(94.1–96.6)	(99.7–99.8)	(87.4–90.9)	(92.0–96.9)	(99.6–99.8)	(85.8–92.2)

*AGA*, appropriate (birthweight 10–90th percentile) for gestational age; *CI*, confidence interval; *LGA*, large (birthweight >90th percentile) for gestational age; *PPV*, positive predictive value; *SGA*, small (birthweight <10th percentile) for gestational age.

*Jelliffe-Pawlowski et al. Dating by metabolic profile: California. Am J Obstet Gynecol 2016*.

a Based on Alexander et al.^48^

**Table 4:** Estimate of week of gestation using metabolic-based algorithm in those identified as preterm^a^

	Training			Testing		
± 1 wk (95% CI)	±2 wk (95% CI)	±3 wk (95% CI)	±1 wk (95% CI)	±2 wk (95% CI)	±3 wk (95% CI)
<37 wk	78.8%	89.8%	95.4%	78.3%	91.7%	96.3%
(78.4–79.2)	(89.5–90.1)	(95.2–95.6)	(77.6–79.0)	(80.7–91.7)	(96.0–96.6)
<32 wk	52.1	72.6	81.7	53.1	73.7	84.9
(50.3–53.9)	(71.1–74.3)	(80.3–83.1)	(49.9–56.3)	(70.9–76.6)	(82.6–87.2)
32–36 wk	80.9	91.2	96.5	80.2	92.5	97.2
(80.5–81.3)	(90.9–91.5)	(96.3–96.7)	(795–80.9)	(92.0–92.9)	(96.9–97.5)

*CI*, confidence interval.

*Jelliffe-Pawlowski et al. Dating by metabolic profile: California. Am J Obstet Gynecol 2016.*

a Where algorithm used linear discriminate for <37 wk (supplementary Table 1) and then, among those positive for <37 wk, used week-specific linear discriminate (Supplementary Table 2, Supplementary Table 3).

**Table 5:** Estimate of population-level preterm birth rates using metabolic-based algorithm^a^

	Training		Testing	
Modeled, % (95% CI)	Fetal ultrasound, % (95% CI)	Modeled, % (95% CI)	Fetal ultrasound, % (95% CI)
<37 wk	7.15	6.11	7.31	6.25
(7.08–7.22)	(6.05–6.17)	(7.19–7.43)	(6.14–6.36)
<32 wk	0.71	0.53	0.71	0.51
(0.69–0.73)	(0.51–0.53)	(0.67–0.75)	(0.48–0.54)
32–36 wk	6.44	5.58	6.60	5.74
(6.37–6.51)	(5.52–5.64)	(6.49–6.71)	(5.63–5.85)

*CI*, confidence interval.

*Jelliffe-Pawlowski et al. Dating by metabolic profile: California. Am J Obstet Gynecol 2016*.

a Where algorithm used linear discriminate for <37 wk (Supplementary Table 1) and then, among those positive for <37 wk, used week-specific linear discriminate (Supplementary Table 2, Supplementary Table 3).
